# Influence of Self-Compassion on the Health of Midwives and Nurses: Protocol for a Scoping Review

**DOI:** 10.2196/21917

**Published:** 2021-03-31

**Authors:** Mitra Javanmard, Mary Steen, Rachael Vernon

**Affiliations:** 1 UniSA Clinical and Health Sciences University of South Australia Adelaide Australia

**Keywords:** self-compassion, self-worth, self-appreciation, self-kindness, midwives, nurses

## Abstract

**Background:**

Self-compassion is recognized to have a positive effect upon a person’s health. However, the influence of self-compassion on the health of midwives and nurses is less well understood. Midwives and nurses often work in highly demanding environments and situations, and are exposed to multiple work-based stressors simultaneously. Stressors such as a demanding clinical workload, high acuity, missing breaks, working more than their contracted hours, insufficient resources and staff, and poor patient outcomes can lead to midwives and nurses feeling physically exhausted and at increased risk of poor mental health. Self-compassion may act as a protective factor, assisting midwives and nurses to remain healthy.

**Objective:**

This scoping review will provide an overview of the evidence base relating to the influence of self-compassion on the health of midwives and nurses.

**Methods:**

The purpose of a scoping review is to comprehensively and systematically review the literature and identify key evidence or gaps. The search strategy for this protocol includes electronic databases such as Medline, Embase, Emcare, PsycInfo, Joanna Briggs Institute, Cochrane Library, and Scopus. Grey literature sources will be also searched, including ProQuest Central, internet search engines (Google Scholar), and manually searched key journals and reference lists of relevant articles. This scoping review will be undertaken in seven stages, guided by established scoping review methods and reporting guidelines: (1) identifying the research questions; (2) identifying relevant studies; (3) selecting the studies; (4) charting the data; (5) collating, summarizing, and reporting the results; (6) consulting; and (7) dissemination of knowledge. Data will be abstracted and presented using the Preferred Reporting Items for Systematic Reviews and Meta-Analyses extension for Scoping Reviews checklist and explanation by three independent researchers.

**Results:**

A preliminary search conducted in Medline (OVID) retrieved 194 results. Completion of the review is expected in December 2020 and will be published in early 2021.

**Conclusions:**

To our knowledge, this will be the first scoping review of evidence-based literature relating to the influence of self-compassion on the health of midwives and nurses. It is anticipated that this analysis of the literature will contribute to understanding how midwives and nurses may use self-compassion in a proactive way to reduce work-based stressors such as burnout, stress, and compassion fatigue. Furthermore, the findings may inform educational needs with implications for clinical practice.

**International Registered Report Identifier (IRRID):**

PRR1-10.2196/21917

## Introduction

### Background

The focus of this scoping review relates to the influence of self-compassion upon midwives and nurses, and how this concept may assist midwives and nurses to remain healthy. According to the World Health Organization (WHO) [1], health is defined as “a state of complete physical, mental and social well-being and not merely the absence of disease or infirmity.” Therefore, health describes more than the mere integrity of the physical body [1]. Physical health and mental health are fundamentally connected and profoundly affect each other [2]. The WHO [3] specifically defines mental health as “a state of well-being in which the individual realizes his or her own abilities, can cope with the normal stresses of life, can work productively and fruitfully, and is able to make a contribution to his or her community.” More recently, Galderisi et al [4] has related mental health to social values and roles:

mental health…enables individuals to use their abilities in harmony with universal values of society. Basic cognitive and social skills; …ﬂexibility and ability to cope with adverse life events and function in social roles.

Self-compassion is a concept of Buddhist philosophy in which a person has the ability to treat oneself with kindness, warmth, and acceptance in times of need [5,6]. Neff [5] and Heffernan et al [7] have described self-compassion as turning compassion inward, and being kind to yourself, and to acknowledge your own humanity, imperfection, and fragility. Neff [5] defined self-compassion as: “being touched by and open to one’s own suffering, not avoiding or disconnecting from it, generating the desire to alleviate one’s suffering and to heal oneself with kindness.”

Three interconnected components have been defined that determine self-compassionate reactions to personal negative emotions and experiences [5,8]: self-kindness versus self-judgment, sense of common humanity versus isolation, and mindfulness versus overidentification. Self-kindness describes an understanding behavior toward oneself in the face of suffering. Common humanity describes the recognition that life stresses and experiences are shared human experiences, rather than an interpretation that they are separate from those of others. Mindfulness describes the balanced awareness of negative thoughts and feelings rather than their overidentification. These individual components are assumed to interact to generate a self-compassionate frame of mind [9]. It has been reported that self-compassion and mindfulness can improve health care professionals’ mental health [10,11] by enhancing optimism [12,13], happiness [14], and by looking at problems from a larger perspective [15].

Understanding and increasing self-compassion in health care professionals continues to be an important yet underrepresented area of research. High rates of work-related stress and burnout in health care professionals [16] can lead to depression, anxiety, sleep disturbances, and fatigue [17]. It has been highlighted that when a person has the ability to have self-compassion, they are more inclined to have good interpersonal relationships and experience a greater sense of happiness when compared to a person who has an impairment in self compassion [18]. Adversely, a lack of self-compassion is associated with psychological distress [19].

Previous studies have suggested that the overall well-being of health care professionals has been influenced by self-compassion [20] by involving self-care behaviors such as allocating time for good nutrition [21], regular outdoor activities and exercise [20-22], and relaxation and spirituality activities [20,21,23]. Importantly, happiness levels can be improved through self-compassion and the use of adaptive behavioral and cognitive tasks [18,24].

Stressors such as a demanding clinical workload, high acuity, missing breaks, working more than their contracted hours, and insufficient resources and staff can lead to midwives and nurses feeling physically and mentally exhausted in the long term [20]. Midwives dealing with pregnant women experiencing stillbirth [25], and oncology nurses looking after patients who are in severe pain, distress, and approaching death [26] are examples of experiences that may lead to negative psychological outcomes. These factors and experiences can have a negative impact on the care given by these midwives and nurses [7].

However, it has been reported that high levels of self-compassion can positively increase overall health and psychological well-being [27-29], whereas low levels of self-compassion are associated with anxiety, stress, and depression [5]. Therefore, self-compassion may act as a protective factor, and help midwives and nurses to remain mentally and physically healthy.

Enhancing self-compassion as a focus of mindfulness education or training has been shown to increase compassionate self-care in health professionals [6,30]. Self-compassion education or training has been recommended for health professionals to help them cope with daily anxiety and stress while providing care, and increasing awareness of the benefits of self-compassion [14]. Therefore, this scoping review will search the literature investigating or exploring the influence of self-compassion for midwives and nurses. It is anticipated that the findings will inform educational needs with implications for self-compassion in clinical practice.

### Aim and Objectives

The initial aim of this scoping review was to identify the influencing factors of self-compassion on midwives. After an initial search, only three studies were found that reported on the influence of self-compassion for midwives [20,31,32]. The published literature appears to have focused mostly on nurses, with a distinct global gap in evidence investigating the influence of self-compassion among midwives. Due to a lack of research studies on midwives, the aim of this scoping review was expanded to include nurses.

The aim of this review is to scope all forms of contemporary literature to determine if there is evidence of self-compassion influencing the health of midwives and nurses. The review objectives are to identify studies that have either investigated or explored factors that influence midwives’ and nurses’ self-compassion, and map factors that positively or negatively impact their health. The review will also identify studies that have directly evaluated education or training programs that focus on self-compassion for midwives and nurses. 

## Methods

### Scoping Review

A scoping review was considered the most appropriate design to address the objectives of this review for several reasons. First, the aim and objectives of this review are broad, and unlike a systematic review or meta-analysis, this scoping review is not trying to answer a specific question but rather to “examine the extent, range, and nature of a research activity” [33]. Second, a scoping review is rigorous, and requires implementation of a comprehensive and systematic approach to searching for relevant literature. Undertaking a scoping review is considered the most appropriate approach to gather evidence from studies using a variety of methodologies, or where there are no reviews being undertaken on the specific topic [34].

This scoping review will primarily use the method established by Arksey and O’Malley [35], incorporating improvements suggested by Levac et al [33] and Peters et al [36], and the adjustments made by Tricco et al [37]. Methodological stages include (1) identifying the research question; (2) identifying relevant studies; (3) selecting studies; (4) charting the data; and (5) collating, summarizing, and reporting the results. Adding two stages, (6) consultation and (7) disseminating the knowledge [33,37], will assist in contextualizing all of the methodological stages utilized to undertake this review. Reporting will be conducted according to the Preferred Reporting Items for Systematic reviews and Meta-Analyses extension for Scoping Reviews (PRISMA-ScR) guidelines [37]. The population, concept, and context (PCC) mnemonic will be used to develop the research questions and search strategy for this scoping review [37].

### Stage 1: Identifying the Research Questions

#### Population

All qualified and registered midwives and nurses who are practicing midwifery and nursing (full time, part time, and casual) will be included in the scoping review. Midwives and nurses in an academic position or undertaking research are also included. Midwives and nurses with dual registration are included. Nursing assistants and supporter workers are not included. Midwifery and nursing students are also excluded. To clarify, a midwife is recognized as

a responsible and accountable professional who works in partnership with women to give the necessary support, care and advice during pregnancy, labour and the postpartum period, to conduct births on the midwife’s own responsibility and to provide care for the newborn and the infant

38

A nurse is defined as “a person who has completed a program of basic, generalized nursing education and is authorized by the appropriate regulatory authority to practice nursing in his/her country” [39].

#### Concept

Self-compassion, generally defined as being kind to yourself, focusing on midwives and nurses and how self-compassion may be a buffer and offer some protective mechanism to remain healthy will be reviewed. The concept of self-compassion comprises six components: self-kindness versus self-judgment, common humanity versus isolation, and mindfulness versus overidentification. The influencing factors of these components upon midwives and nurses will be reviewed. The focus of this scoping review is specifically related to self-compassion and its components; therefore, other subject headings such as self-care, self-efficacy, or empathy will not be reviewed.

#### Context

The scoping review will consider studies that involved qualified and registered midwives and nurses undertaken in any health care setting, including, but not restricted to, hospitals and community, medical, and educational centers. Research involving self-compassion education for midwives and nurses will be reviewed. The original Self-Compassion Scale (SCS) is a 26-item questionnaire and widely used self-report measure developed to assess the six components of self-compassion [40]. Furthermore, a shorter version (12 items) of the SCS has been developed [41]. Therefore, studies that have utilized validated measures for self-compassion will be included. The effectiveness of self-compassion measurement scales, and education or training provided for qualified midwives and nurses will be reported. 

#### Formulating the Final Research Questions

According to Arksey and O’Malley [35], this scoping review will follow an iterative process for developing the research questions. Initially, broad research questions were conceived to ensure that the review will identify the diversity and scope of the literature available. However, as this review continues, the research questions may be reformulated and new questions may appear over time. The final questions are: (1) Is there evidence of specific factors associated with self-compassion upon midwives’ and nurses’ health status? (2) How have the included studies reported on the measurement and effectiveness of self-compassion? (3) Is there evidence to support self-compassion education or training for midwives and nurses, and is this associated with improved health outcomes? (5) Have studies reported any challenges or limitations upon implementation of self-compassion education or training for midwives and nurses?

The aim of this scoping review will comprehensively address these broad research questions; however, key elements will conceptualize the review aim and objectives to guide the search strategy.

### Stage 2: Identifying Relevant Studies

#### Types of Studies

This review will consider all types of studies, including quantitative, qualitative, and mixed-methods studies. All types of quantitative studies will be included (eg, cross-sectional studies, correlational studies, randomized controlled trials [RCTs], non-RCTs, single-case studies, and case-control studies). Furthermore, all types of qualitative studies will be included (eg, ethnography, phenomenology, grounded theory) and mixed methods (eg, convergent, exploratory, and explanatory sequential and advanced designs). Other types of studies such as systematic reviews and scoping reviews will also be considered. To be included in the review, studies will need to consider at least one component of self-compassion measured with a self-compassion scale among midwives and nurses.

Self-compassion scales utilized in reviews and conference abstracts will be reviewed and reported separately in the Discussion section. Only results and findings of primary studies will be reported in the Results section. Letters to the editor, commentaries, case reports, and ongoing studies are also excluded. The review is limited to papers written in English. Due to the increasing focus on self-compassion in the last two decades in western society, this scoping review will include relevant studies published from 2000 to 2020.

#### Databases

Specific keywords and controlled vocabulary terms will be used to maximize sensitivity and specificity within the search. The search strategy will aim to find both published and unpublished English-language studies. The search strategy was developed in Medline via the Ovid platform. The details of the search in Medline are presented in Multimedia Appendix 1. The search will be expanded to include the Embase, Emcare, PsycInfo, Joanna Briggs Institute (Ovid), Scopus, and Cochrane Library (Wiley) databases to identify articles on this topic, followed by analysis of the text words contained in titles and abstracts, and index terms used to describe these articles. The following database sources will be searched to identify any grey literature: ProQuest Central and Google/Google Scholar, and the bibliographies of relevant studies and reviews. Additionally, the reference lists of eligible studies will be sourced. This approach will inform the development of a search strategy, including identified keywords and index terms that will be adapted for each information source. As the focus of this scoping review is on self-compassion, MeSH (Medical Subject Heading) terms such as self-care, self-efficacy, or empathy will not be reviewed. A search strategy is provided in Table 1.

**Table 1:** Draft search terms.

Category	Search terms
Participants	Midwives OR midwi* OR nurses OR nurs*
Concept	Self-compassion* OR Self?compassion OR Self-kindness* OR self?kindness OR Self-worth* OR self?worth OR Self-appreciation* OR self?appreciation
Context	Health care facilities (eg, community and medical centers and hospitals) Educational centers (eg, universities)
Study types	Quantitative, qualitative, and mixed-methods research studies

The complete and final search strategy will be provided in a follow-up publication. Searches will also be rerun prior to the final analyses to retrieve any subsequent studies that meet the inclusion criteria. If additional sources are identified while the review is being performed, the inclusion of these sources will be documented. Upon completion, the results from each database will also be documented.

### Stage 3: Study Selection

All references from the initial main search will be exported to Covidence (a web-based source selection tool) for eliminating duplicates, screening of titles and abstracts, and full-text reviews. After excluding duplicates, two reviewers will screen the title and abstract for each of the papers according to the proposed eligibility criteria. To refine the web-based source selection tool, pilot testing of a random sample of 25 titles/abstracts will be undertaken by the authors. Any discrepancies will be discussed and modifications will be made [42]. The full-text version of potential suitable papers will then be read and screened by two reviewers against the inclusion/exclusion criteria. A separate appendix will be submitted for excluded sources and the reasons for exclusion will be stated using Covidence [41]. Consensus will be reached by agreement.

If there are any cases of disagreement between the two reviewers, which cannot be resolved through discussion, a third independent reviewer will be asked to assess the paper(s) in question and consensus will be reached by agreement. The selection process will be reported using a PRISMA-ScR flow diagram [37].

### Stage 4: Charting the Data

Data charting will be used for extracting data from included studies in scoping reviews [33,35,36]. A standardized data extraction spreadsheet template will be developed using Microsoft Excel to extract data from all papers meeting the inclusion criteria. Data will be charted by one reviewer and checked by another reviewer. Any discrepancies will be resolved by consensus of the research team. Pilot testing of 5-10 full-text articles for data charting will be undertaken to ensure that key information is extracted completely [42]. Data extraction will be an iterative process, incorporating an initial trial of data charting and team consultation throughout the process to ensure consistency with the review purpose and questions, and to include new questions that may arise during the process. Regular communication between reviewers will facilitate this process and ensure the reliability of data charting. This approach will be based on providing the data necessary for addressing the main objective of this review; that is, to identify factors that influence midwives’ and nurses’ self-compassion, and map factors that positively or negatively impact their health. At present, these categories include (but are not limited to): bibliographic information of the study, study aims, research design, setting/context, number of participants, measure(s) used to assess the influential factors of self-compassion, self-compassion education or training (if applicable), analyses conducted, results of statistical analyses, and summary of findings. Furthermore, the authors of this scoping review will provide their own interpretations regarding conclusions, strengths, limitations, and recommendations of included studies. A draft of the data extraction tool is presented in Table 2. This draft will be modified and revised as necessary during the process of extracting data from each included study.

**Table 2:** Relevant studies.

Data	Details to be extracted (if available)
Publication summary	Author(s), year, location, title, aim(s), type of study
Settings	Hospital, university, etc
Population	Total sample size
Self-compassion scales	The Neff Self-Compassion Scales 24-item; Short Version SCS 12-item
Other used scales (if applicable)	Copenhagen Burnout Inventory 19-item to measure burnout; Perceived Stress Scale 10-item to measure stress; Professional Quality of Life Scale, version 5 to measure compassion satisfaction
Factors associated with self-compassion	Burnout, stress, and compassion satisfaction, etc
Self-compassion education or training (if applicable)	Any used scales
Outcomes	Any identified factors affected by self-compassion
Others	Conclusions, strengths and limitations, recommendations

### Stage 5: Collating, Summarizing, and Reporting the Results

The purpose of this scoping review is to collate research findings and present an overview of all material reviewed, rather than a systematic synthesis of evidence that provides results on a narrowly defined question. Stage 5 of the review will be completed in three steps: analyzing (collating and summarizing) the data, reporting results, and applying meaning to the results.

The first step will involve analyzing quantitative data, and the results of individual studies will be analyzed and tabulated. Any qualitative data will be analyzed thematically and reported either narratively or tabulated. Additionally, further narrative description of all results will be provided to aid interpretation of the findings as they relate to the research questions. The tables will report on distribution of studies by type, year of publication, country of origin, research methods, all utilized scales, factors associated with self-compassion, and findings.

For reporting results, a narrative summary, represented by categories, will describe how the results relate to the review aim and objectives. The results will be classified in main conceptual categories that will be obtained during the results extraction. Specific factors that influenced completeness and usefulness will be grouped by domain, and the final list of factors will be determined and agreed on by all reviewers. The final synthesis of the reviewed literature will be presented in tables, graphs, or charts, complemented by a narrative description.

The final step will apply meaning to the results, and a narrative descriptive approach will be used to highlight gaps in existing research evidence, and consider the implications of findings within the broader context such as research, policy, and practice.

### Stage 6: Consulting

The consultation stage will be used to add methodological rigor, and to ensure applicability and usability of the results [33]. To reach this goal, the reviewers will share preliminary findings from stage 5 (collating, summarizing, and reporting results) with an independent self-compassion researcher/author and a midwife or a nurse to validate the findings. The feedback will be taken into consideration and integrated within the overall review findings.

### Stage 7: Disseminating the Knowledge

According to the framework suggested by Levac et al [33] and Tricco et al [37], we consider it important to make the content of this scoping review available to key stakeholders (ie, midwives and nurses), with the goal of increasing awareness of the findings and facilitate evidence-informed decision making. Following the scoping review, the reviewers will disseminate the findings several ways by authoring a journal publication, an editorial conference presentation, and a written summary report for professional midwifery and nursing organizations and social media outlets.

## Results

A preliminary search performed in Medline retrieved 194 results. It is anticipated that all stages of the review will be completed in December 2020 and results will be disseminated through peer-review publications in early 2021.

## Discussion

In the general population, self-compassion is associated with positive health outcomes. However, the influence of self-compassion on midwives’ and nurses’ health is less well known. Midwives and nurses are often exposed to multiple work-based stressors; thus, the influence of self-compassion on their health may be an important factor that has implications for future practice. This review will provide an evidence-based overview of the influence or potential influence of self-compassion on midwives’ and nurses’ health. The findings of this scoping review will identify gaps in the current literature on self-compassion research, and highlight related priorities for future research for midwives and nurses. The findings may have practical implications by identifying specific self-compassion components that can be implemented in future self-compassion education or training. One of the strengths of the proposed review is to identify if self-compassion education or training programs can potentially improve the awareness and ability of midwives and nurses to have self-compassion. We anticipate that the outcomes of this review will be relevant to midwives, nurses, researchers, clinical management, stakeholders, and commissioners on an international scale.

## Appendix

Multimedia Appendix 1 Search strategy for MEDLINE (2000 to present).

Multimedia Appendix 2 External peer-review report.

## Abbreviations

PRISMA-ScR: Preferred Reporting Items for Systematic Reviews and Meta-Analyses extension for Scoping Reviews
RCT: randomized controlled trial
SCS: Self-Compassion Scale
WHO: World Health Organization
