# Supplementary figures and images for: Influence of Self-Compassion on the Health of Midwives and Nurses: Protocol for a Scoping Review
Source: JMIR Res Protoc. 2021 Mar 31;10(3):e21917. doi: 10.2196/21917 (PMC8047817; doi:10.2196/21917)

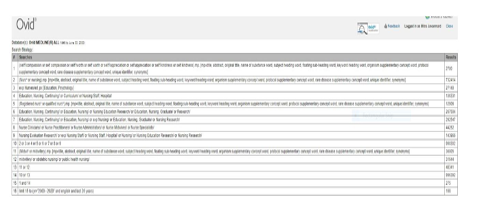

Supplement: Multimedia Appendix 1 [file resprot_v10i3e21917_app1.png]
