# Supplementary material for: Influence of Self-Compassion on the Health of Midwives and Nurses: Protocol for a Scoping Review
Source: JMIR Res Protoc. 2021 Mar 31;10(3):e21917. doi: 10.2196/21917 (PMC8047817; doi:10.2196/21917)
Supplement: Multimedia Appendix 2 [file resprot_v10i3e21917_app2.docx]

**Existing Peer-review Reports - Reviewers’ Feedback**

**Reviewer 1.**

Firstly, I would congratulate you on producing such a good protocol. It is well written and of a very high standard. So, for the most part, I only had a few very trivial comments (which are added as track changes in the attached protocol). I thought the description of your proposed scoping review method was very good, and matches expected best practice for scoping reviews. However, I have a few comments on the concept of self-compassion and on the review’s mental and physical health focus.

There is a focus in the review questions and the review title on mental and physical health, but in the protocol the information about that focus is limited to a single sentence in the ‘Type of Studies Section’. Mental and physical health are very broad concepts, so I suspect a little more detail is needed to explain what that means in practical terms in the context of your scoping review.

My major comment is about the concept of ‘self-compassion’. This is a very broad concept, one that is not easily defined or categorised. But the section titled ‘Concept’ provides only a limited description. I note in the ‘Types of Studies section’, a little more information is provided (but that seems to be in the wrong place). So, my first comment is I think you need to group all the information about self-compassion into the section titled ‘Concept’, to better describe what it means. When you are undertaking the scoping review, will you only include studies that use the term ‘self-compassion’, or will you have a broader understanding of what constitutes self-compassion for nurses and midwives. So as a second comment, I think more detail is needed to better define what self-compassion means in practical terms in the context of your scoping review.

I hope these comments help.

**Reviewer 2.**

The topic is relevant and doing a scoping review will provide insights into the benefits or not of nurses and midwives having self-compassion. This will also provide an evidence-base for further research.

This scoping review protocol is overall well written, and the proposed methodology is explained very clearly. The methodology relates to the latest methods used for scoping reviews and a strength is how you clearly explain the evolving process and stages to be undertaken.

However, I would recommend some further details is required re: explaining what self-compassion is and relate this to nurses and midwives. I would recommend refer to Kristen Neff’s work and include a definition.

Additionally, I would recommend focusing on mental health problems and not mental and physical health, as this is very broad. Focussing on mental health will provide a more specific focus and is in alignment with the aim and objectives of the scoping review.

I recommend the inclusion of a definition for mental health such as the WHO one as this would be helpful for the reader.

| **Amendments** | | |
| --- | --- | --- |
| **Reviewer 1** | | |
| Mental and physical health are very broad concepts. A little more detail is needed to explain what that means in practical terms in the context of your scoping review. | Physical health has been deleted. Focus is on mental health. | Title and whole the manuscript  pp. 3-4 ‘Introduction’ |
| Concept of ‘self-compassion’ is a very broad concept. I think more detail is needed to better define what self-compassion means in practical terms in the context of your scoping review. | Self-compassion and its interconnected components have been defined. | P.3  ‘Introduction’ |
| **Reviewer 2** |  |  |
| Further details are required re: explaining what self-compassion is and relate this to nurses and midwives. I would recommend refer to Kristen Neff’s work and include a definition. | Amended. | pp. 3-4 ‘Introduction’ |
| I would recommend focusing on mental health problems and not mental and physical health, as this is very broad. | Amended.  Physical health has been deleted. | Title and whole the manuscript |
| I recommend the inclusion of a definition for mental health such as the World Health Organization one. | Amended. | P.3  ‘Introduction’ |
